# Effectiveness and Immunogenicity of the MMR Vaccine Against SARS-CoV-2 Among Healthcare Workers

**DOI:** 10.3390/v17020215

**Published:** 2025-02-01

**Authors:** Hyeri Seok, Joon-Yong Bae, Jooyun Kim, Won Suk Choi, Heedo Park, Jungmin Lee, Sohyun Lee, Chulwoo Kim, Man-Seong Park, Dae Won Park

**Affiliations:** 1 Division of Infectious Diseases, Department of Internal Medicine, Korea University Ansan Hospital, Ansan 15355, Republic of Korea; hyeri.seok@gmail.com (H.S.); jooyuna777@gmail.com (J.K.); cmscw@hanmail.net (W.S.C.); 2 Department of Microbiology, Institute for Viral Diseases, Vaccine Innovation Center, Korea University Medicine, Seoul 02841, Republic of Korea; harbe3103@korea.ac.kr (J.-Y.B.); pdh0919@korea.ac.kr (H.P.); 2019010546@korea.ac.kr (J.L.); leesh9253@korea.ac.kr (S.L.); kimcw@korea.ac.kr (C.K.)

**Keywords:** SARS-CoV-2, COVID-19, measles–mumps–rubella vaccine, vaccine effectiveness, vaccine immunogenicity

## Abstract

The purpose of this study was to evaluate the effectiveness and immunogenicity of the measles–mumps–rubella (MMR) vaccine against SARS-CoV-2 in healthcare workers at one medical institution. The effectiveness of the MMR vaccine against SARS-CoV-2 was evaluated in overall healthcare workers (HCWs). In addition, neutralizing antibodies to SARS-CoV-2 were measured according to the subjects’ measles immunity status with serum samples collected before the coronavirus disease 2019 pandemic period. The effectiveness of the MMR vaccine for SARS-CoV-2 in all HCWs and measles IgG-positive subjects was 34% (adjusted odds ratio [aOR] = 1.20, 95% confidence interval [CI] = 0.53–2.70) and 34% (aOR = 0.66, CI = 0.38–18.4), respectively. The neutralizing antibody levels for SARS-CoV-2 were low in all groups regardless of the measles immune status. The MMR vaccine alone may not provide sufficient protection against SARS-CoV-2.

## 1. Introduction

Coronavirus disease 2019 (COVID-19) is a current pandemic infectious disease, and vaccine coverage has been accomplished quickly worldwide [1]. In August 2024, 13.64 billion people were vaccinated worldwide (https://data.who.int/dashboards/covid19/vaccines/; accessed on 22 August 2024), and in Korea, 45 million people were vaccinated as of September 2023 (https://coronaboard.kr/en/; accessed on 22 August 2024). With the limited availability of antiviral agents against COVID-19, high vaccination rates have reduced the morbidity and severity of COVID-19 [1,2].

At the time of emergence of COVID-19, when the vaccination rate was low, the susceptibility to COVID-19 differed from person to person; however, data on host susceptibility to COVID-19 were not readily available. Some live vaccines, such as the measles–mumps–rubella (MMR) vaccine or Bacille Calmette–Guérin (BCG) vaccine, have been reported to have protective effects against SARS-CoV-2 through nonspecific immunity after live vaccination [3,4,5,6,7,8,9]. The hypothesis suggests that the measles virus and SARS-CoV-2 virus have a 32% structural similarity in their spike glycoproteins, and some parts of the SARS-CoV-2 glycoprotein may interfere with the fusion protein F1 of the measles vaccine [9]. Additionally, vaccine-induced interferon-gamma and NK cell activation may enhance natural immunity against SARS-CoV-2 through cross-reactivity [5,10]. Subsequent studies have reported that the effect of the MMR vaccine on SARS-CoV-2 remains controversial [3]. Most studies analyzed clinical data and vaccination history, and further studies with laboratory data are still needed.

In Korea, when small outbreaks occurred in Korea, healthcare workers (HCWs) were required to have their immune status evaluated, and our hospital also evaluated the overall immune status of HCWs for measles [11]. We could also determine the immunogenicity of SARS-CoV-2 through the sample. Thus, we conducted this study to evaluate the effectiveness and immunogenicity of the MMR vaccine against SARS-CoV-2 in HCWs at one medical institution.

## 2. Methods

### 2.1. Immune Status for Measles and MMR Vaccination

The Korea University Ansan Hospital assessed the susceptibility of all HCWs to measles, following an executive order due to a measles outbreaks in Ansan, Gyeonggi-do. Out of 1278 HCWs who were tested for measles virus IgG, 1202 had positive results for IgG, 21 were equivocal, and 55 (4.3%) had negative results. Population was classified into three groups based on measles immunity: the natural immunity group (Group 1) included MeV-IgG-seropositive subjects with a history of infection or those born before 1968; the vaccine-induced immunity group (Group 2) comprised MeV-IgG-seropositive individuals who received a primary measles vaccination, had no history of measles infection, and were born after 1968; and the vaccine failure group (Group 3) consisted of MeV-IgG seronegative subjects who had received a primary measles vaccination [12]. Of the subjects identified as susceptible to measles, belonging to Group 3, 34 subjects were given two doses of the MMR vaccine, administered 4 weeks apart.

### 2.2. Evaluation of the Effectiveness and Immunogenicity of the MMR Vaccine Against SARS-CoV-2

Since the first confirmed case of COVID-19 occurred in South Korea on 20 January 2020, the COVID-19 outbreak spread nationwide. All medical institutions were required to conduct exposure investigations after exposure to patients with COVID-19, SARS-CoV-2 PCR testing for exposed HCWs, and active surveillance. Based on the measles immunity status of HCWs surveyed in 2019 and the results of the SARS-CoV-2 PCR testing performed from January 2020 to March 2022, we evaluated the effectiveness and immunogenicity of the MMR vaccine against COVID-19. The plaque reduction neutralization test (PRNT) was used to detect functional neutralizing antibodies, which is a gold standard method for evaluating measles immunogenicity [13]. To determine the neutralizing antibody titer, PRNT was performed using SARS-CoV-2 isolated in Korea (BetaCoV/Korea/KCDC03/2020, National Culture Collection for Pathogens [NCCP;43326]). Two-fold serial dilutions of heat-inactivated (30 min at 56 °C) serum samples were mixed with an equal amount of virus suspension containing approximately 100 plaque-forming units (PFUs) in 0.1 mL. After incubating the mixture at 37 °C for 1 h (h), the mixture (0.2 mL) was used to inoculate Vero cells for the plaque assay. After incubating the plate at 37 °C for 1 h, virus-infected Vero cells were overlaid with agar and incubated at 37 °C for 3 days, followed by staining of the cells to visualize the plaques and counting of the plaques to measure the PRNT_50_. The PRNT_50_ titer was calculated as the highest serum dilution that showed a 50% reduction in the number of viral plaques compared with that of a phosphate-buffered saline-treated control. The values of PRNT_50_ were interpreted as follows: less than 1:10 indicates low immunity, between 1:10 and 1:80 indicates moderate immunity, and 1:80 or higher indicates high immunity. All experiments involving SARS-CoV-2 were conducted with the approval of the Institutional Biosafety Committee of Korea University (IBC 2021-0020) and were performed in the Biosafety Level 3 (BSL-3; KCDC-18-302) facility at the Korea University College of Medicine.

## 3. Statistical Analyses

Multivariate logistic regression was used to evaluate the vaccine effectiveness. Vaccine effectiveness was defined as 1 minus the odds of vaccination in cases, divided by the odds of vaccination in controls. All statistical tests were two-tailed, and *p*-values ≤ 0.05 were considered statistically significant. All statistical analyses were performed using SPSS Statistics version 20.0 for Windows (IBM Corp., Armonk, NY, USA).

## 4. Results

**Figure 1:**
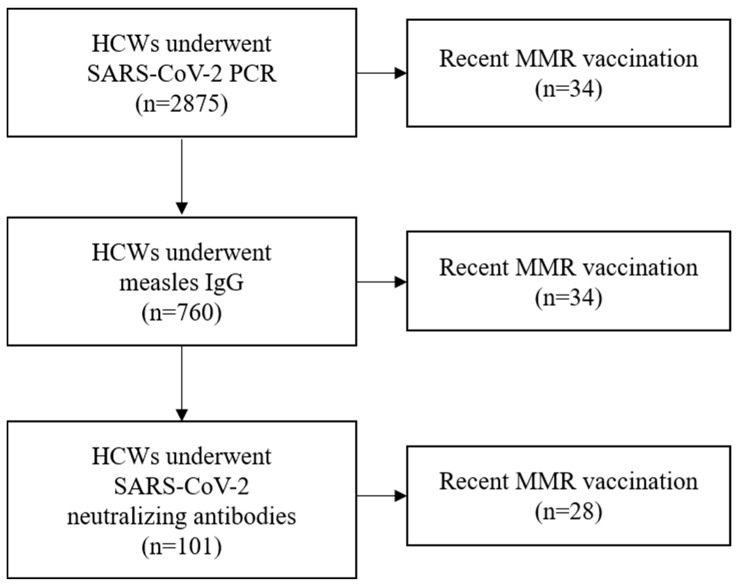
Study population in this study. Abbreviation: HCWs, healthcare workers; MMR, measles–mumps–rubella.

During the study, 2875 HCWs underwent SARS-CoV-2 PCR testing due to the presence of symptoms or for exposure investigation for COVID-19. Among these 2875 subjects, 620 were positive for SARS-CoV-2 on PCR. To evaluate immunogenicity against measles, ELISA testing was performed in 760 subjects. A total of 216 subjects with confirmed measles antibodies were positive for SARS-CoV-2 PCR. We measured neutralizing antibodies to SARS-CoV-2 in 101 blood samples with measurable neutralizing antibodies for measles in 2019. The year 2019 was a time before the advent of COVID-19; therefore, there was no effect of COVID-19 disease or vaccination at all. The subject numbers of the three groups classified according to measles immunity were 33, 40, and 28, respectively. Figure 1 shows the flowchart of study population.

Table 1 shows the MMR vaccine effectiveness against SARS-CoV-2 based on the subjects who were vaccinated with the MMR vaccine and had a SARS-CoV-2 PCR test. The vaccine effectiveness in the vaccinated group compared with the unvaccinated group was 34% (adjusted odds ratio [aOR] = 1.20, 95% confidence interval [CI] = 0.53–2.70). When the effectiveness of the MMR vaccine was evaluated in the group in which measles IgG was identified, the vaccine effectiveness remained 34% (aOR = 0.66, CI = 0.38–18.4) (Table 2).

**Figure 2:**
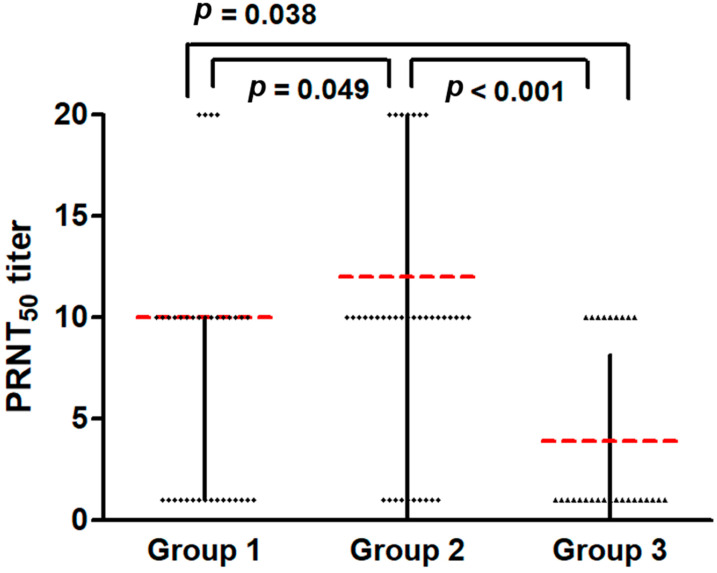
Comparison of SARS-CoV-2 neutralizing antibody titers in each group according to measles immunity status. Group 1: natural immunity; Group 2: vaccine-induced immunity; Group 3: vaccine failure. Abbreviation: SARS-CoV-2, severe acute respiratory syndrome coronavirus 2; PRNT_50_, 50% plaque reduction neutralization test. The red lines and black dotted lines are the median and interquartile range values of PRNT_50_ titer in each group.

The neutralizing antibody results for SARS-CoV-2 according to measles immunity status are presented in Table 3. The median SARS-CoV-2 neutralizing antibodies for PRNT50 were 1:10, 1:10, and 1:1 in Groups 1, 2, and 3, respectively. All groups showed below low-level neutralizing antibody titers. Nevertheless, Group 3, which included sub-jects susceptible to measles, showed statistically significantly lower neutralizing antibodies titers against SARS-CoV-2 (Figure 2). There was no difference in the neutralizing antibody titers against SARS-CoV-2 between Group 1 and Group 2.

## 5. Discussion

This study evaluated the effectiveness and immunogenicity of the MMR vaccine against SARS-CoV-2, which is a live vaccine other than COVID-19 vaccines. Unlike previous studies, this study is significant in that it evaluated the effectiveness and immunogenicity of the MMR vaccine by measuring neutralizing antibodies against SARS-CoV-2 using blood samples collected before the advent of COVID-19 [3,4,5,6,7]. Based on the results of this study, the MMR vaccine alone may not provide a sufficient protective effect against SARS-CoV-2.

The previous hypothesis that live vaccines, including the MMR vaccine, would be effective against SARS-CoV-2 is based on the following mechanisms. Live vaccines have trained immunity, which leads to nonspecific immune effects in the process of mimicking natural infection [6]. Trained immunity is activated in the innate immunity stage, not in the adaptive immunity stage; this enhances the immune response and production of pro-inflammatory cytokines through epigenetic and metabolic reprogramming. Another hypothesis is that the structural similarity of one or more components of the MMR vaccine to the epitope of SARS-CoV-2 recognized by the immune system may lead to cross-reactivity with other antigens [9]. In a previous study, seven various cross-reactive epitopes were found between SARS-CoV-2 and the MMR vaccine [14]. Another study reported that COVID-19 and MMR vaccination shared a T-cell population including a memory T-cell subset [5]. In clinical studies, a measles IgG titer was associated with decreased severity of COVID-19 [7]. The MMR vaccinated population showed a low incidence and severity of COVID-19 [4].

In this study, the MMR vaccine did not show a significant protective effect against SARS-CoV-2, with a vaccine effectiveness of 34%. These data suggest that the MMR vaccination alone does not provide sufficient protection against SARS-CoV-2. However, the measles susceptible group in this study was too small to prove this hypothesis; thus, an effect of the sample size limit to obtain statistically significant values cannot be completely ruled out. This is because Korea has been verified as country that has eliminated measles by the World Health Organization (WHO), and most of the population has acquired immunity against measles.

In terms of vaccine immunogenicity, neutralizing antibodies against SARS-CoV-2 in all groups classified according to measles immunogenicity showed values below the low level. In Group 2, the neutralizing antibody titers were significantly higher than those in the other groups; however, it should be noted that the absolute titers were still low. A prior study reported that measles IgG titers were increased significantly in patients with COVID-19 compared with a control group and that measles IgG showed a statistically significant correlation with nucleocapsid protein among SARS-CoV-2 antibodies [15]. This suggests that measles immunogenicity unrelated to MMR vaccination may be associated with B-cell cross-reactivity against SARS-CoV-2. Another hypothesis is that the overall B-cell function of the subjects in Group 3 may be decreased; however, this must be identified through the evaluation of B-cell function.

The limitations of this study are as follows. First, this study included a period when the COVID-19 pandemic had already started and COVID-19 vaccination had begun among the study population; thus, interference of the pandemic and vaccination cannot be completely excluded. However, data from the early stage of the pandemic were included, and all HCWs refrained from outside activities as much as possible during the pandemic period and focused only on work in the hospital; therefore, the impact of community COVID-19 infections other than hospital infections would have been minimized. Second, this study was conducted at a single institution. Nevertheless, in that all HCWs at one medical institution were evaluated, the data may represent relatively homogeneous data in terms of age, gender, etc. Finally, although we included as many populations as possible to evaluate vaccine effectiveness, few subjects were assessed in the measurement of neutralizing antibodies. This is limiting as past blood samples could not be retrospectively collected; therefore, only samples collected during previous measles outbreaks were used. In addition to measuring neutralizing antibodies, further studies on the evaluation of T-cell immunity or B-cell function may be required.

In conclusion, MMR vaccination and immunogenicity against measles did not provide sufficient protection against SARS-CoV-2. Although the MMR vaccine may be partially effective against SARS-CoV-2, replacing the COVID-19 vaccine with the nonspecific immunity of the MMR vaccine is not recommended when a sufficient supply of the COVID-19 vaccine is available.

## Figures and Tables

**Table 1:** MMR vaccine effectiveness against SARS-CoV-2 in overall HCWs.

	SARS-CoV-2 (+) *		SARS-CoV-2 (−) *		Unadjusted OR	Unadjusted Vaccine Effectiveness	Adjusted OR	Adjusted Vaccine Effectiveness
	*n*	%	*n*	%	OR (95% CI)	% (95% CI)	OR (95% CI)	% (95% CI)
Recent MMR vaccination	9	1.5	25	1.1	1.27 (0.57–2.85)	N/A	1.20 (0.53–2.70)	34 (0–47)
Control	611	98.5	2230	98.9				

* SARS-CoV-2 (+) indicates a positive SARS-CoV-2 PCR test result, while (−) indicates a negative SARS-CoV-2 PCR test result.

**Table 2:** MMR vaccine effectiveness against SARS-CoV-2 in HCWs with measles IgG.

	SARS-CoV-2 (+) *		SARS-CoV-2 (−) *		Unadjusted OR	Unadjusted Vaccine Effectiveness	Adjusted OR	Adjusted Vaccine Effectiveness
	*n*	%	*n*	%	OR (95% CI)	% (95% CI)	OR (95% CI)	% (95% CI)
Recent MMR Vaccination	9	4.2	25	4.6	0.90 (0.41–1.96)	10 (0–59)	0.66 (0.38–1.84)	34 (0–62)
Control	207	95.8	519	95.4				

* SARS-CoV-2 (+) indicates a positive SARS-CoV-2 PCR test result, while (−) indicates a negative SARS-CoV-2 PCR test result.

**Table 3:** Immunogenicity of measles and SARS-CoV-2 according to measles immunity status.

	Group 1 (*n* = 33, Natural Immunity)	Group 2 (*n* = 40, Vaccine-Induced Immunity)	Group 3 (*n* = 28, Vaccine Failure)	*p* Value	*p* Value (Group 1 vs. 2)	*p* Value (Group 1 vs. 3)	*p* Value (Group 2 vs. 3)
Age	52 ± 1 (51–53)	41 ± 5 (38–46)	32 ± 6 (27–35)	<0.001	<0.001	<0.001	<0.001
Male	15 (37.5)	5 (12.5)	11 (27.5)	0.039	0.029	0.796	0.069
Median SARS-CoV-2 neutralizing Ab, PRNT_50_ titer	10 (1–10)	10 (7.75–10)	1 (1–10)	<0.001	0.049	0.038	<0.001

## Data Availability

The data presented in this study are available on request from the corresponding author due to privacy and ethical reasons.
